# “Input/output cytokines” in epidermal keratinocytes and the involvement in inflammatory skin diseases

**DOI:** 10.3389/fimmu.2023.1239598

**Published:** 2023-10-10

**Authors:** Shin Morizane, Tomoyuki Mukai, Ko Sunagawa, Kota Tachibana, Yoshio Kawakami, Mamoru Ouchida

**Affiliations:** ^1^ Department of Dermatology, Okayama University Graduate School of Medicine, Dentistry and Pharmaceutical Sciences, Okayama, Japan; ^2^ Department of Immunology and Molecular Genetics, Kawasaki Medical School, Kurashiki, Japan; ^3^ Department of Molecular Oncology, Okayama University Graduate School of Medicine, Dentistry and Pharmaceutical Sciences, Okayama, Japan

**Keywords:** epidermal keratinocytes, input cytokines, output cytokines, biologics, inflammatory skin diseases

## Abstract

Considering the role of epidermal keratinocytes, they occupy more than 90% of the epidermis, form a physical barrier, and also function as innate immune barrier. For example, epidermal keratinocytes are capable of recognizing various cytokines and pathogen-associated molecular pattern, and producing a wide variety of inflammatory cytokines, chemokines, and antimicrobial peptides. Previous basic studies have shown that the immune response of epidermal keratinocytes has a significant impact on inflammatory skin diseases. The purpose of this review is to provide foundation of knowledge on the cytokines which are recognized or produced by epidermal keratinocytes. Since a number of biologics for skin diseases have appeared, it is necessary to fully understand the relationship between epidermal keratinocytes and the cytokines. In this review, the cytokines recognized by epidermal keratinocytes are specifically introduced as “input cytokines”, and the produced cytokines as “output cytokines”. Furthermore, we also refer to the existence of biologics against those input and output cytokines, and the target skin diseases. These use results demonstrate how important targeted cytokines are in real skin diseases, and enhance our understanding of the cytokines.

## 1 Introduction

In recent years, many biologics targeting cytokines have been clinically used for inflammatory skin diseases. Therefore, we must understand the importance of cytokines in the pathogenesis of the diseases. It is widely known that cytokines mainly function among immunocytes such as lymphocytes, but in fact, epidermal keratinocytes, which are resident cells, also recognize and produce various cytokines.

Epidermal keratinocytes occupy 90% or more of the epidermis, form a physical barrier (1). On the other hand, epidermal keratinocytes also form an innate immunological barrier with the potential to mount an innate immune response. For example, epidermal keratinocytes also express a variety of cytokine receptors, and microbial sensors such as Toll-like receptor (TLR) 1, TLR2, TLR3, TLR4, TLR5, TLR6, TLR9, MDA5 (melanoma differentiation-associated gene 5) and RIG-I (retinoic acid-inducible gene-I) (2–6). Also, epidermal keratinocytes are capable of producing inflammatory cytokines and chemokines (5, 6). In addition, the cells show antibacterial activity by expressing antibacterial peptides such as defensins, cathelicidin, and S100 proteins (7). Through these immunological functions, epidermal keratinocytes play an important role in the pathogenesis of inflammatory skin diseases including atopic dermatitis (AD), psoriasis, several pustular dermatoses and so on (8–10).

The purpose of this review is to provide foundation of knowledge on the cytokines which are recognized or produced by epidermal keratinocytes. Since a number of biologics for skin diseases have appeared, it is necessary to fully understand the relationship between epidermal keratinocytes and the cytokines. We here focus on pro- or anti-inflammatory cytokines except growth factors in epidermal keratinocytes. The cytokines directly recognized by epidermal keratinocytes are specifically introduced as “input cytokines”, and the produced cytokines as “output cytokines”. Furthermore, we also refer to the existence of biologics against those input and output cytokines and the target skin diseases. Some of these biologics have already been approved and are in use, while others have not been shown to be effective. Recognizing these findings will enhance our understanding of the cytokines.

## 2 “Input cytokines” in epidermal keratinocytes

**Figure 1:**
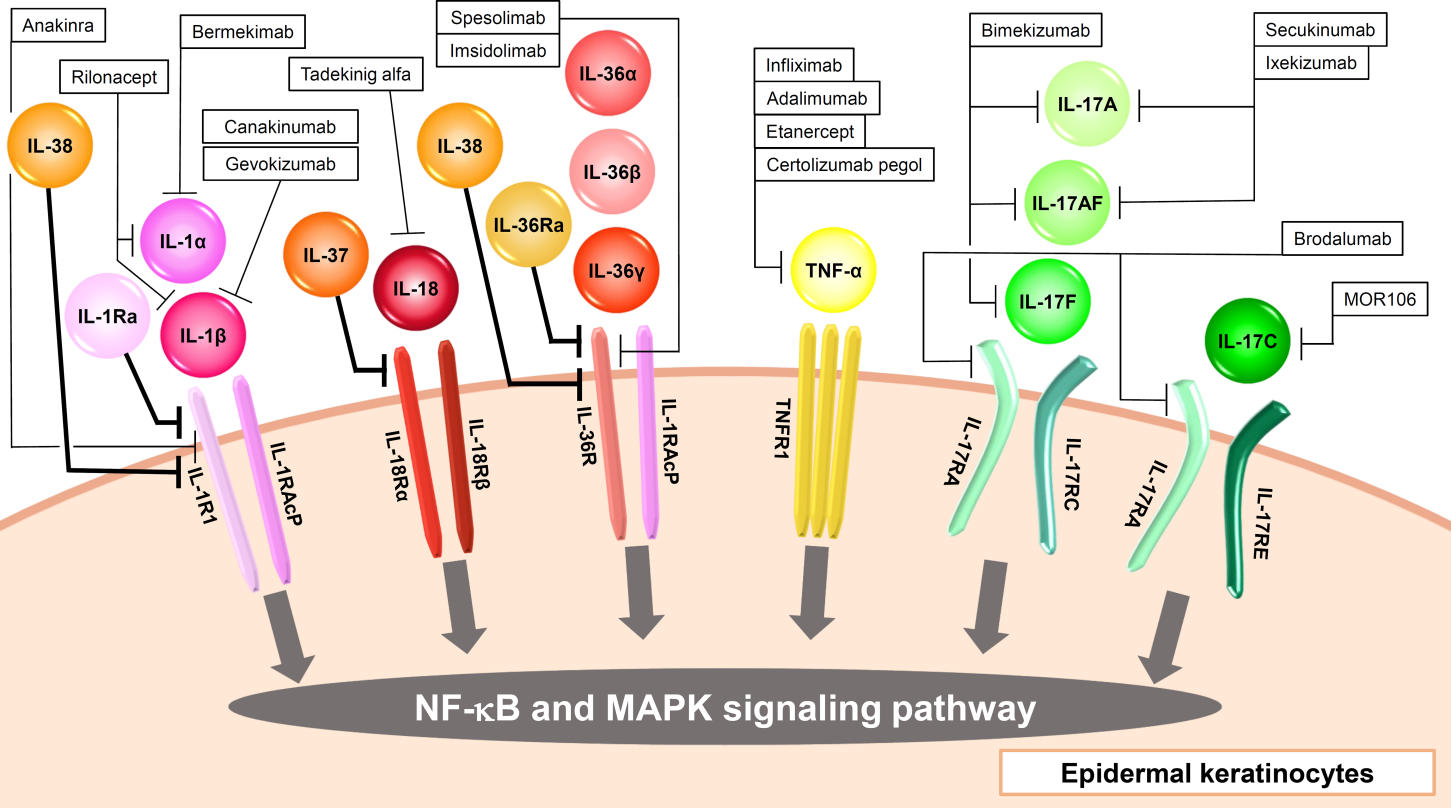
Input cytokines which activate NF-κB and MAPK signaling pathway in epidermal keratinocytes.

**Figure 2:**
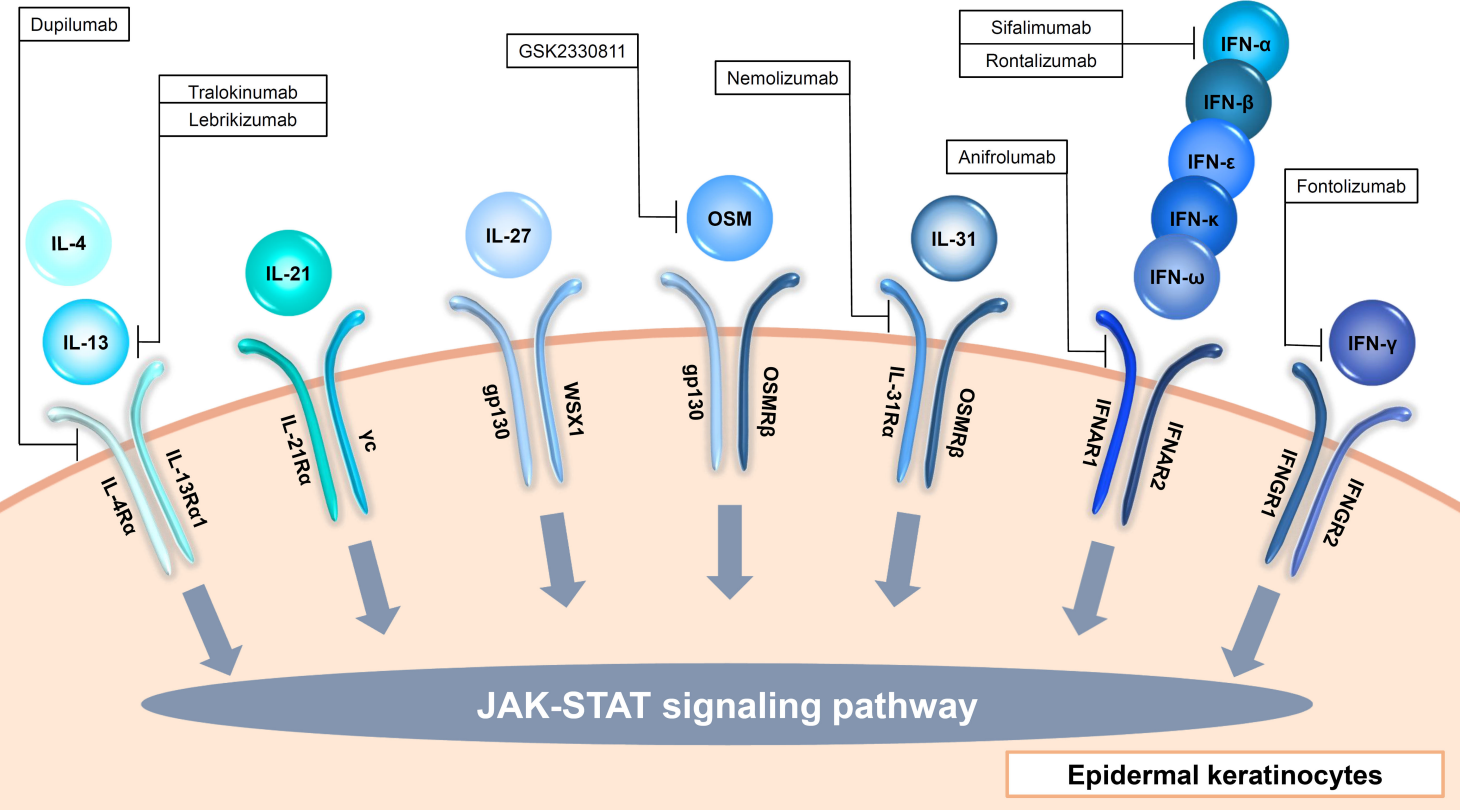
Input cytokines which activate JAK-STAT signaling pathway in epidermal keratinocytes (γc cytokines, IL-6 family cytokines, type I IFNs and type II IFN).

**Figure 3:**
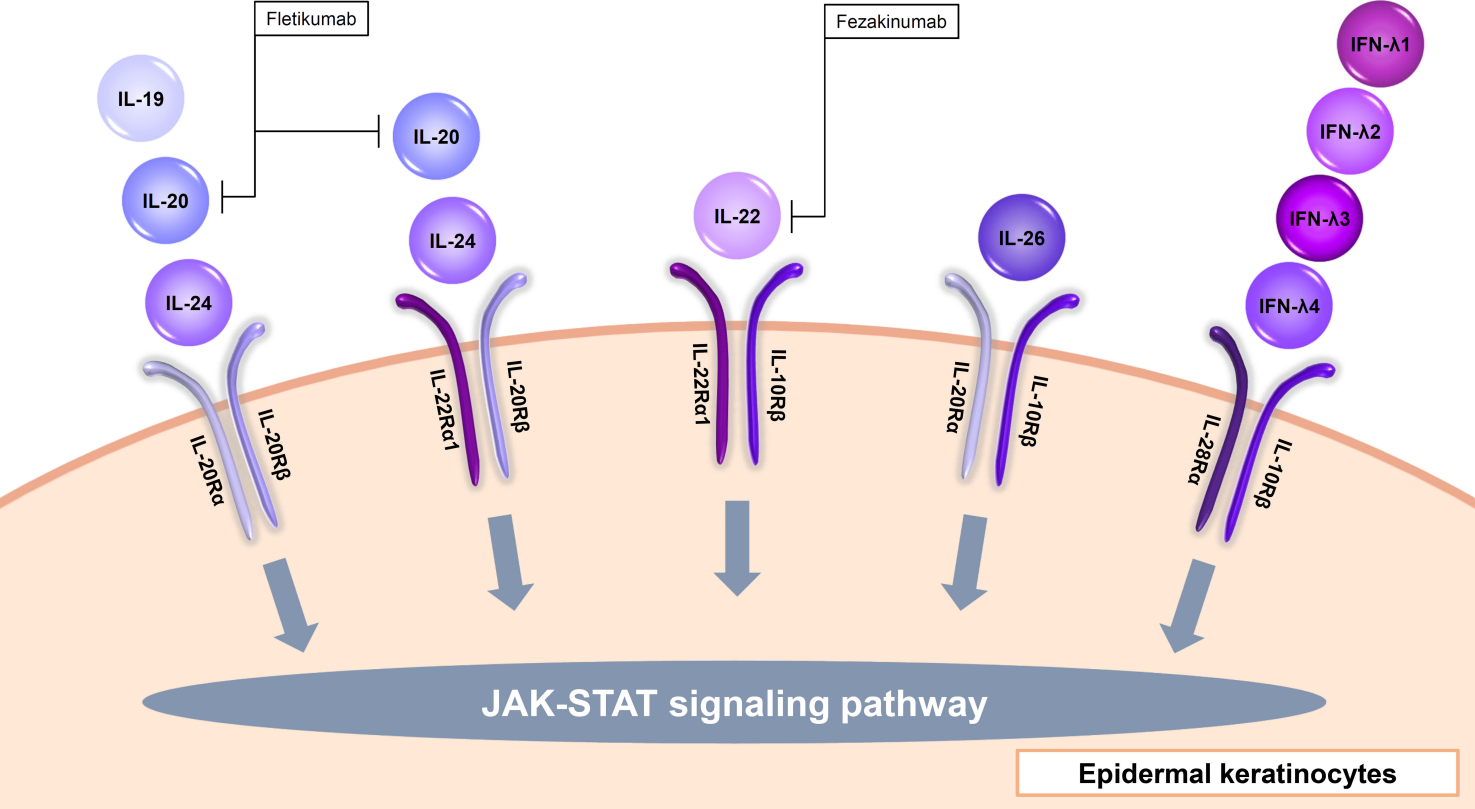
Input cytokines which activate JAK-STAT signaling pathway in epidermal keratinocytes (IL-20 family cytokines and type III IFNs).

“Input cytokines” in epidermal keratinocytes include IL-1α/β/Ra, IL-4, IL-13, IL-17A/AF/C/F, IL-18, IL-19, IL-20, IL-21, IL-22, IL-24, IL-26, IL-27, IL-31, IL-36α/β/γ/Ra, IL-37, IL-38, IFN-α/β/ε/γ/κ/λ1/λ2/λ3/λ4/ω, oncostatin M (OSM) and TNF-α (**Figures 1**–**3**, **Table 1**).

**Table 1:** Input cytokines in epidermal keratinocytes.

Cytokine	Classification	Receptor	Signaling
IL-1α/β	IL-1 family	IL-1R1/IL-1RAcP	NF-κB and MAPK
IL-1Ra	IL-1 family	IL-1R1	Act as IL-1R antagonist
IL-4	γc family	IL-4Rα/IL-13Rα1	JAK-STAT
IL-13	IL-4 like cytokine	IL-4Rα/IL-13Rα1	JAK-STAT
IL-17A/AF/F	IL-17 family	IL-17RA/IL-17RC	NF-κB and MAPK
IL-17C	IL-17 family	IL-17RA/IL-17RE	NF-κB and MAPK
IL-18	IL-1 family	IL-18Rα/IL-18Rβ	NF-κB and MAPK
IL-19	IL-20 family	IL-20Rα/IL-20Rβ	JAK-STAT
IL-20/24	IL-20 family	IL-20Rα/IL-20Rβ, IL-22Rα1/IL-20Rβ	JAK-STAT
IL-21	γc family	IL-21Rα/γc	JAK-STAT
IL-22	IL-20 family	IL-22Rα1/IL-10Rβ	JAK-STAT
IL-26	IL-20 family	IL-20Rα/IL-10Rβ	JAK-STAT
IL-27	IL-6 family	gp130/WSX1	JAK-STAT
IL-31	IL-6 family	IL-31Rα/OSMRβ	JAK-STAT
IL-36α/β/γ	IL-1 family	IL-36R/IL-1RAcP	NF-κB and MAPK
IL-36Ra	IL-1 family	IL-36R	Act as IL-36R antagonist
IL-37	IL-1 family	IL-18Ra	Act as IL-18R antagonist
IL-38	IL-1 family	IL-1R1 or IL-36R	Act as IL-1R or IL-36R antagonist
IFN-α/β/ε/κ/ω	Type I IFN	IFNAR1/IFNAR2	JAK-STAT
IFN-γ	Type II IFN	IFNGR1/IFNGR2	JAK-STAT
IFN-Λ1/Λ2/Λ3/Λ4	Type III IFN	IL-28Rα/IL-10Rβ	JAK-STAT
OSM	IL-6 family	gp130/OSMRβ	JAK-STAT
TNF-α	TNF family	TNFR1	NF-κB and MAPK

### 2.1 IL-1 family cytokines

The IL-1 family consists of 11 cytokines which are further divided into inflammatory cytokines with agonistic activity (IL-1α, IL-1β, IL-18, IL-33, IL-36α, IL-36β, IL-36γ) and anti-inflammatory cytokines with antagonistic activity (IL-1Ra, IL-36Ra, IL-37, IL-38) (11). They are also classified into three subfamilies (IL-1, IL-18, IL-36 subfamily) according to their structures and receptors (11). Most of the human IL-1 family cytokine genes are located on chromosome 2, and the IL-18 and IL-33 genes are located on chromosomes 11 and 9, respectively (12). Among them, epidermal keratinocytes recognize IL-1α, IL-1β, IL-18, IL-36α, IL-36β, IL-36γ, IL-1Ra, IL-36Ra, IL-37, and IL-38 (**Figure 1**, **Table 1**).

IL-1α and IL-1β bind to IL-1R1, and this binding signals via TIR-MyD88, leading to NF-κB and MAPK activation (**Figure 1**, **Table 1**) (13). IL-1RAcP is the co-receptor for IL-1R1 (**Figure 1**, **Table 1**). IL-1-bound IL-1RI associates with IL-1RAcP to form a heterodimer. Signal transduction requires the presence of IL-1RI and IL-1RAcP molecules. IL-1α is produced as precursors and activated by calpain (10). IL-1β is also produced as precursors and activated by casepase-1. IL-1α and IL-1β induce inflammatory cytokines including TNF-α and IL-6, and chemokines including IL-8 in epidermal keratinocytes (14). IL-1Ra binds to IL-1R1, and this cytokine work as the inhibitor of IL-1α and IL-1β (**Figure 1**, **Table 1**) (13).

IL-1 signaling is thought to play an important role in not only autoinflammatory diseases but also various inflammatory skin diseases. Therefore, it has attracted attention as a therapeutic target. Anakinra, a recombinant IL-1Ra which blocks the activities of the proinflammatory cytokines IL-1α and IL-1β, is clinically used for rheumatoid arthritis (RA), neonatal-onset multisystem inflammatory disease (NOMID), cryopyrin-associated periodic syndromes (CAPS), systemic juvenile idiopathic arthritis (sJIA), adult-onset Still disease (AOSD), Schnitzler’s Syndrome (SS), and deficiency of IL-1RA (DIRA) (15). However, it was not significantly effective in a phase II randomized, double-blind clinical trial for palmoplantar pustulosis (PPP) (16). On the other hand, another group reported that anakinra up to 300mg daily showed positive responses with localized and generalized pustular psoriasis (GPP) in a phase II open-label trial (17). Anakinra has also been used in hidradenitis suppurativa (HS) by several groups with controversial results (10). Rilonacept is an IL-1 receptor fusion protein consisting of the Fc portion of human IgG1 and the human IL-1 receptor which traps both IL-1α and IL-1β, and clinically used for familial cold autoinflammatory syndrome (FCAS), Muckle–Wells syndrome (MWS), and recurrent pericarditis (15). A clinical trial for cold contact urticaria (CCU) is currently ongoing with this agent (18). Canakinumab is a human anti-IL-1β monoclonal antibody, and clinically used for FCAS, MWS, CAPS, familial Mediterranean fever (FMF), mevalonate kinase deficiency (MKD), tumor necrosis factor receptor-associated periodic syndrome (TRAPS), and AOSD (15). Canakinumab has also shown contradictory efficacy results in HS (10). In an open-label prospective study, this agent was effective for pyoderma gangrenosum (PG) (10). Bermekimab, a human anti-IL-1α monoclonal antibody, showed efficacy in phase II open-label studies in HS patients (10). Gevokizumab is a humanized anti-IL-1β monoclonal antibody, and clinical trials for PG are currently ongoing with this agent (18).

IL-18 binds to IL-18Rα, and this binding signals via TIR-MyD88, leading to NF-κB and MAPK activation (**Figure 1**, **Table 1**) (13). IL-18Rβ is the co-receptor for IL-18Rα (**Figure 1**, **Table 1**). Like IL-1 signaling, the signal transduction requires the heterodimerization of IL-18Rα and IL-18Rβ (**Figure 1**, **Table 1**) . IL-18 is produced as precursors and activated by casepase-1 (10). Epidermal keratinocytes express IL-18Rα and IL-18Rβ. When IL-18 binds to these receptors on the surface of keratinocytes, it triggers a signaling cascade within the cells, leading to various cellular responses such as the induction of CXCL9, CXCL10, CXCL11, major histocompatibility complex (MHC) class I, and MHC class II expression (19, 20). IL-18 is considered to be involved in the pathogenesis of psoriasis, AD, and AOSD, and tadekinig alfa, a human recombinant IL-18-binding protein, is currently investigated in a phase II open-label clinical trial on patients with AOSD (18).

IL-36α, IL-36β, and IL-36γ bind to IL-36R, and these binding signal via TIR-MyD88, leading to NF-κB and MAPK activation (**Figure 1**, **Table 1**) (13). IL-1RAcP is the co-receptor for IL-36R (**Figure 1**, **Table 1**). Like IL-1 and IL-18 signaling, the signal transduction requires the heterodimerization of IL-36R and IL-1RAcP (**Figure 1**, **Table 1**). IL-36 cytokines are produced as precursors and activated by neutrophil-derived proteases (10). Similar to IL-1α and IL-1β, IL-36α, IL-36β, and IL-36γ induce TNF-α, IL-6, IL-8, G-CSF, GM-CSF, CXCL1, CXCL10, CCL20, and RANTES in epidermal keratinocytes (21, 22).

IL-36Ra binds to IL-36R, and this cytokine works as the inhibitor of IL-36α, IL-36β, and IL-36γ (**Figure 1**, **Table 1**) (13). Deficiency of IL-36Ra develop GPP, which suggests the importance of IL-36 signaling in the disease (23). In fact, spesolimab, a humanized anti-interleukin-36 receptor monoclonal antibody which blocks human IL-36α-, IL-36β-, and IL-36γ-induced IL-36R activation, show significant clinical improvement in GPP (24). Additional studies of spesolimab are currently being performed in patients with PPP and HS (10). Imsidolimab is also a humanized anti-interleukin-36 receptor monoclonal antibody which blocks human IL-36α-, IL-36β-, and IL-36γ-induced IL-36R activation, and clinical trials for HS and GPP are currently ongoing with this agent (10).

IL-37 is an anti-inflammatory cytokine, and reported to suppress the production of CXCL8, IL-6, and S100A7 which are induced by the mixture of five proinflammatory cytokines in human keratinocyte cell line HaCaT cells (25). Extracellularly, IL-37 binds to IL-18Ra and recruits IL-1R8 to form the IL-37/IL-1R8/IL-18Ra complex, inhibiting IL-18R-dependent inflammation (10).

IL-38 is also anti-inflammatory cytokine, and reported to inhibit IL-36γ-induced inflammatory molecules in epidermal keratinocytes (26). IL-38 binds to IL-1RAcP or IL-36R, and works as the inhibitor of IL-1α/β or IL-36α/β/γ, respectively (10).

### 2.2 Gamma chain cytokines

The γc cytokines family consists of IL-2, IL-4, IL-7, IL-9, IL-15, and IL-21, and among them, IL-4 and IL-21 are input cytokines in epidermal keratinocytes (**Figure 2**, **Table 1**) (27). IL-13, another type 2 cytokine which shares IL-4Rα and IL-13Rα1 with IL-4, is also an input cytokine in the cells (**Figure 2**, **Table 1**) (28). Th2 cells release IL-4 and IL-13, and type 2 innate lymphoid cells produce IL-13 (29). The IL-4 and IL-13 signaling in the cells decrease the expression of filaggrin, loricrin, an involucrin via JAK-STAT pathway (30, 31). These cytokines also suppress ceramide synthesis and inhibit the expression of elongases which lengthen fatty acid chain in the cells (32–34). In addition, antimicrobial peptides expression is also suppressed by IL-4 and IL-13 in the cells, which enhances the susceptibility to infection (35). Furthermore, IL-4 and IL-13 increase serine protease KLK7 expression and function in the cells (36). Recently, these cytokines were also reported to impair TLRs-mediated barrier functions in the early phases of AD (37). These findings suggest that IL-4 and IL-13 contribute to not only allergic inflammation but also barrier dysfunction. The importance of IL-4 and IL-13 in skin diseases is found in recent biologics. Anti-IL-4Rα antibody dupilumab which blocks both IL-4 and IL-13 signaling and anti-IL-13 antibody including tralokinumab and lebrikizumab show clinical efficacy in AD (38–41). In addition, dupilumab represents significant improvement in prurigo nodularis (PN) (42).

IL-21 is produced by NKT and CD4(+) T cells, and signals via JAK-STAT pathway (**Figure 2**, **Table 1**) (43). IL-21R is up-regulated in patients with systemic sclerosis (SSc) and might be involved in the pathogenesis of SSc via induction of VEGF (44). IL-21 is also highly expressed in the skin of individuals with psoriasis, and stimulates epidermal keratinocytes to proliferate and causes epidermal hyperplasia (45).

### 2.3 IL-6 family cytokines

The IL-6 family consists of 11 cytokines and shares 130-kDa signal-transducing β-receptor subunit gp130, except IL-31 (46–48). All the cytokines activate JAK-STAT signaling pathway. Among them, IL-27 induces CXCL9, CXCL10, CCL2, CCL5, and enhance anti-viral activity in epidermal keratinocytes (49–51). Another IL-6 family member, OSM, is also recognized via gp130 and OSM receptor beta (OSMRβ) by epidermal keratinocytes (**Figure 2**, **Table 1**) (52). OSM is produced by T cells, monocytes, macrophages, hepatocytes and endothelial cells (52). OSM is involved with innate immunity, angiogenesis, adhesion, motility, tissue remodeling, cell cycle and transcription in epidermal keratinocytes (52, 53). Since this cytokine show synergy with TNF-α, IL-1α, IL-17A, and IL-22 in production of antimicrobial peptides, it is considered to be involved in pathogenesis of psoriasis (53). OSM is also implicated in the pathogenesis of SSc, and a randomized phase 2 study ofan anti-OSM monoclonal antibody GSK2330811 in SSc was conducted. However, its effects were not different from placebo (54). IL-31 is also an input cytokine which signals through heterodimeric receptors composed of the OSMRβ and the interleukin 31 receptor alpha (IL-31Rα) (**Figure 2**, **Table 1**) (48). IL-31 is mainly produced by Th2 cells, and suppresses the skin barrier protein expression such as filaggrin and involucrin and induces the expression of several chemokines in epidermal keratinocytes (55, 56). IL-31Rα is also expressed in sensory nerves and IL-31 promotes nerve fiber extension, suggesting that IL-31 is involved in pruritus in AD (57). Actually, nemolizumab, a humanized monoclonal antibody against IL-31Rα which blocks signaling from IL-31, provides improvement of pruritis in patients with AD in a 16-week, double-blind, phase 3 trial (58).

### 2.4 IL-17 family cytokines

The IL-17 family consists of 6 homodimers IL-17A to IL-17F and 1 heterodimer IL-17AF (59). On the other hand, the IL-17 receptor family consists of 5 molecules, IL-17RA-RE (59). IL-17RA is a common receptor and forms heterodimeric complexes with IL-17RB, IL-17RC and IL-17RE. Epidermal keratinocytes recognize IL-17A, IL-17C, IL-17F, and IL-17AF and then strongly produce inflammatory cytokines, chemokines, and antimicrobial peptides (**Figure 1**, **Table 1**) (60). IL-17A, IL-17AF, and IL-17F are mainly produced by Th17 cells, and share the heterodimeric receptor of IL-17RA and IL-17RC (**Figure 1**, **Table 1**) (59). Binding of these cytokines to their receptors recruits Act1 to which TRAF6 binds. TAK1-NF-κB and MAPK-AP1 axes are activated downstream of TRAF6. IL-17C are produced by epithelial cells rather than immune cells, and binds to the heterodimeric receptor of IL-17RA and IL-17RE, and shows similar activation to IL-17A (**Figure 1**, **Table 1**). However, the ability to induce inflammation in epidermal keratinocytes is reported to be stronger in the order of IL-17A, IL-17AF, IL-17F, and IL-17C (60). These cytokines, especially IL-17A, are considered to play a critical role in the pathogenesis of psoriasis, and anti-IL-17A antibody including secukinumab and ixekizumab, anti-IL-17A/IL-17F antibody bimekizumab, and anti-IL-17RA antibody brodalumab which blocks the signaling of IL-17A, IL-17A/F, IL-17F, IL-17C, and IL-17E, show high clinical efficacy in psoriasis (61–64). Secukinumab and bimekizumab are also reported to be clinically effective in HS (65, 66).

### 2.5 IL-20 family cytokines

The IL-20 family consists of IL-19, IL-20, IL-22, IL-24, IL-26, and type III IFNs. IL-19, IL-20, and IL-24 signal through the IL-20Rα/IL-20Rβ heterodimer. Furthermore, IL-20 and IL-24 also signal through the IL-22Rα1/IL-20Rβ heterodimer (**Figure 3**, **Table 1**) (67). IL-19, IL-20, and IL-24 is mainly produced by myeloid cells but can also be produced by epidermal keratinocytes (68). TNF-α and IFN-γ enhance IL-20Rα expression in the cells (69). These cytokines all induce epidermal keratinocytes to proliferate and to express inflammatory and immunomodulatory mediators through activation of STAT3 (67). IL-20 was considered to be involved in the pathogenesis of psoriasis, and a phase I study with an anti-IL-20 monoclonal antibody fletikumab for psoriasis was conducted, however the study was terminated due to lack of efficacy (70).

IL-22 is known to exert protective functions in barrier defense, tissue repair, and homeostasis depending on the context, in various organs including the skin (71). Epidermal keratinocytes recognize IL-22 through the IL-22Rα1 and IL-10Rβ heterodimer (**Figure 3**, **Table 1**) (72). IL-22 is mainly produced by Th1, Th17, and Th22 cells and also type 3 innate lymphoid cells (72–74). IL-22 up-regulates, in a dose-dependent manner, the expression of S100A7, S100A8, S100A9, a group of proinflammatory molecules belonging to the S100 family of calcium-binding proteins, as well as the matrix metalloproteinase 3, the platelet-derived growth factor A, and the CXCL5 chemokine (75). IL-22 also down-regulates the expression of genes associated with keratinocyte differentiation such as filaggrin (75). In addition, IL-22 strongly induces hyperplasia of reconstituted human epidermis (75). Therefore, IL-22 is considered to contribute to the acanthosis in psoriasis and lichenification in AD. However, the inhibitors of and IL-22 (fezakinumab) did not show sufficient improvement in psoriasis (70). On the other hand, fezakinumab, anti-IL-22 antibody, showed clinical efficacy in moderate-to-severe AD (76).

IL-26 is an input cytokine in epidermal keratinocytes. IL-26 is produced mainly by Th1, Th17, or natural killer cells (77, 78). IL-26R is a heterodimer composed of two receptor proteins: IL-20Rα and IL-10Rβ (**Figure 3**, **Table 1**) (79). IL-26 enhances the production of FGF1, FGF2, and FGF7 from epidermal keratinocytes and vascular endothelial cells (80). These may promote angiogenesis in patients with T cell-mediated skin inflammation, including psoriasis (80). IL-26 enhanced IL-8, IL-1β, CCL20, IL-33, and β-defensin 2 expression via JAK1, JAK2, and TYK2 in normal human epidermal keratinocytes (81). These may be involved in the pathogenesis of AD (81).

### 2.6 type I interferons

Type I interferon (IFN) members consist of IFN-α, IFN-β, IFN-ϵ, IFN-κ and IFN-ω, and bind to the heterodimeric receptor of IFN-α/β receptor 1(IFNAR1) and 2(IFNAR2), resulting in the activation of JAK1 and non-receptor tyrosine kinase 2 (TYK2) and the formation of STAT1-STAT2-IRF9 complex which is called ISGF3 (82). Almost all cell types produce type I IFNs (82). Since epidermal keratinocytes express both IFNAR1 and IFNAR2, the cells recognize type I IFNs (**Figure 2**, **Table 1**) (83). For example, IFN-κ induces IFN-κ expression itself and enhances the anti-viral activity against HSV-1 in epidermal keratinocytes (84). In addition, IFN-α and IFN-κ increase IL-6 production in the cells, which is considered to be associated with the pathogenesis of cutaneous lupus erythematosus (85).

### 2.7 type II interferon

Epidermal keratinocytes also recognize Type II IFN, IFN-γ which is produced by T cells, B cells, NK cells, NKT cells, and dendritic cells (86). IFN-γ binds to the heterodimeric IFN-γ receptor (IFNGR) complex comprising IFNGR1 and IFNGR2 (**Figure 2**, **Table 1**) (86). The signal phosphorylates and activates JAK1, JAK2, and STAT1, which leads to the homodimerization of STAT1 (86). Stimulation with IFN-γ in epidermal keratinocytes increases terminal differentiation of cells, inhibits proliferation, and enhance anti-viral activities (87, 88). Furthermore, IFN-γ cooperates with TNF-α and IL-17A to induce the production of cytokines, chemokines, and antimicrobial peptides (89–91). IFN-γ is increased in the skin lesions of psoriasis, and the disease was previously considered to be a Th1 disease. Therefore, a clinical trial with humanized anti–IFN-γ antibody (Fontolizumab) for moderate-severe plaque psoriasis was performed, however, no significant clinical changes were observed (92).

### 2.8 type III interferons

Type III IFNs, including IFN-λ1 (IL-29), IFN-λ2 (IL-28A), IFN-λ3 (IL-28B), and IFN-λ4, are involved in inhibiting viral infection similar to type I IFNs (93, 94). Type III IFNs act via the heterodimer of IL-28Rα and IL-10Rβ (**Figure 3**, **Table 1**) (93). These cytokines are input cytokines in epidermal keratinocytes, for example, IFN-λ1 is shown to enhance anti-viral activity through an increase in TLR3 in the cells (95).

### 2.9 tumor necrosis factor

Epidermal keratinocytes recognize TNF-α. TNF-α was discovered as a necrotic cytokine in solid tumors and later turned out to be a major cytokine involved in inflammation (96). TNF-α is produced from almost all cells, and are thought to exist both upstream and downstream of the pathological cascade of various inflammatory diseases. Since epidermal keratinocytes express TNFR1 receptors and produce TNF-α, autocrine phenomena are observed and an inflammatory loop is formed (**Figure 1**, **Table 1**) (97). The importance of TNF-α in various skin diseases is easily found in the clinical use of anti-TNF-α antibodies against the diseases. TNF-α inhibitors including infliximab, adalimumab, etanercept, and certolizumab pegol are clinically effective in psoriasis (8). Infliximab and adalimumab also show clinical efficacy in HS, and PG (98–101).

## 3 “Output cytokines” in epidermal keratinocytes

**Figure 4:**
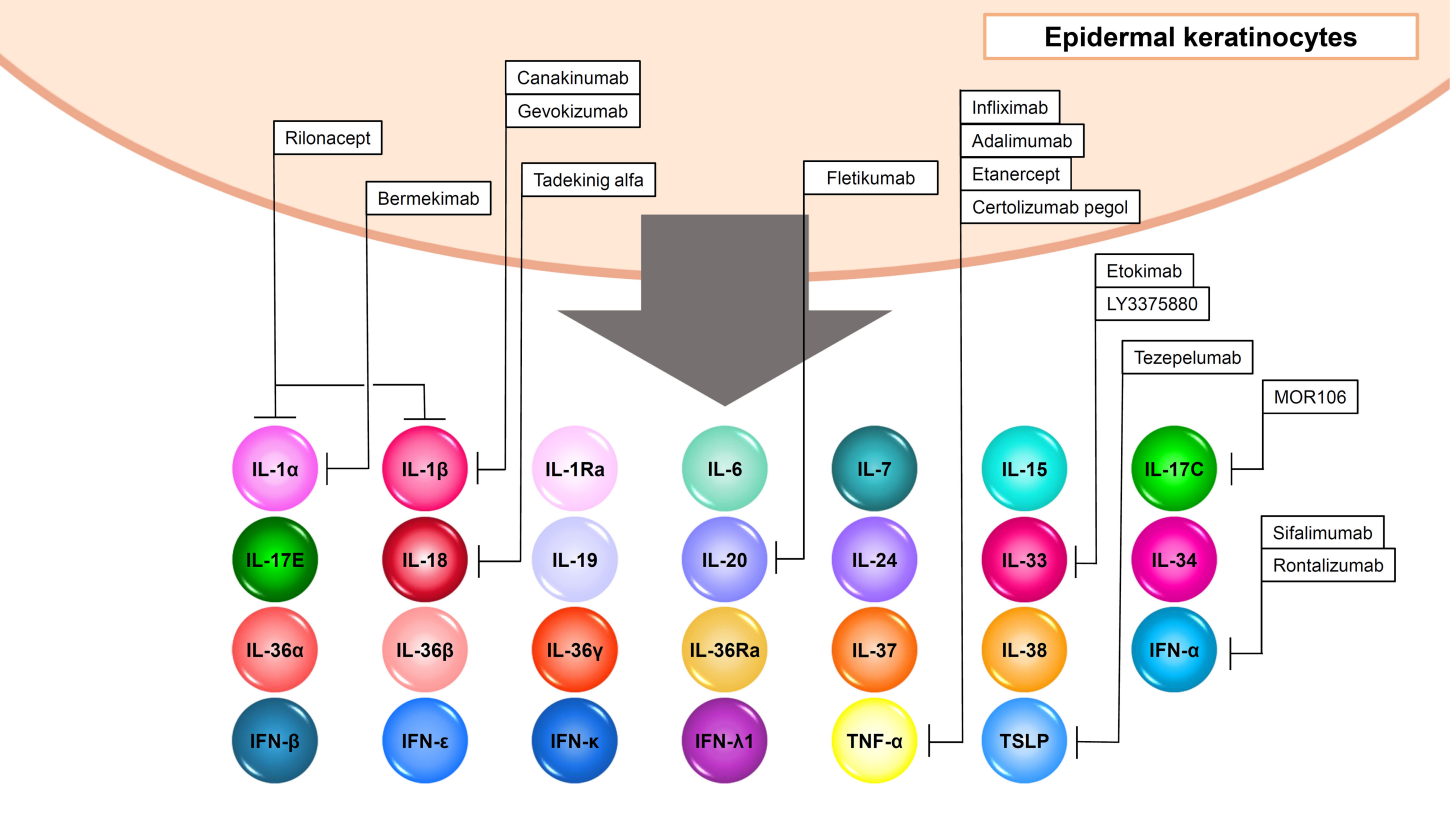
Output cytokines in epidermal keratinocytes.

“Output cytokines” in epidermal keratinocytes include IL-1α/β/Ra, IL-6, IL-7, IL-15, IL-17C, IL-17E (IL-25), IL-18, IL-19, IL-20, IL-24, IL-33, IL-34, IL-36α/β/γ/Ra, IL-37, IL-38, IFN-α/β/ε/κ/λ1, thymic stromal lymphopoietin (TSLP), and TNF-α (**Figure 4**, **Table 2**).

**Table 2:** Output cytokines in epidermal keratinocytes.

Cytokine	Classification	Receptor	Signaling
IL-1α/β	IL-1 family	IL-1R1/IL-1RAcP	NF-κB and MAPK
IL-1Ra	IL-1 family	IL-1R1	Act as IL-1R antagonist
IL-6	IL-6 family	IL-6R/gp130	JAK-STAT
IL-7	γc family	IL-7Rα/γc	JAK-STAT
IL-15	γc family	IL-2/15Rβ/γc	JAK-STAT
IL-17C	IL-17 family	IL-17RA/IL-17RE	NF-κB and MAPK
IL-17E	IL-17 family	IL-17RA/IL-17RB	NF-κB and MAPK
IL-18	IL-1 family	IL-18Rα/IL-18Rβ	NF-κB and MAPK
IL-19	IL-20 family	IL-20Rα/IL-20Rβ	JAK-STAT
IL-20/24	IL-20 family	IL-20Rα/IL-20Rβ, IL-22Rα1/IL-20Rβ	JAK-STAT
IL-33	IL-1 family	ST2/IL-1RAcP	NF-κB and MAPK
IL-34	CSF-1-like	CSF-1R Syndecan-1 PTP-ζ	JAK-STAT NF-κB and MAPK, Caspase, AMPK/ULK1, PI3K/AKT
IL-36α/β/γ	IL-1 family	IL-36R/IL-1RAcP	NF-κB and MAPK
IL-36Ra	IL-1 family	IL-36R	Act as IL-36R antagonist
IL-37	IL-1 family	IL-18Ra	Act as IL-18R antagonist
IL-38	IL-1 family	IL-1R1 or IL-36R	Act as IL-1R or IL-36R antagonist
IFN-α/β/ε/κ	Type I IFN	IFNAR1/IFNAR2	JAK-STAT
IFN-Λ1	Type III IFN	IL-28Rα/IL-10Rβ	JAK-STAT
TNF-α	TNF family	TNFR1	NF-κB and MAPK
TSLP	IL-7-like cytokine	TSLPR/IL-7Rα	JAK-STAT

### 3.1 IL-1 family cytokines

Epidermal keratinocytes produce inflammatory IL-1 family cytokines with agonistic activity including IL-1α, IL-β, IL-18, IL-33, IL-36α, IL-36β, and IL-36γ and anti-inflammatory IL-1 family cytokines with antagonistic activity including IL-1Ra, IL-36Ra, IL-37, and IL-38 (**Figure 4**, **Table 2**). Since IL-1α, IL-β, IL-18, IL-36α, IL-36β, and IL-36γ are also input cytokines and are capable of inducing themselves, inflammation loops are formed in the cells (**Figures 1**, **4**, **Tables 1**, **2**) (14, 102).

IL-33 is an IL-1-family cytokine that is over-expressed in the keratinocytes of patients with AD (103, 104). IL-33 is also in an activated state in the precursor and is rather inactivated when cleaved by caspase-1 or caspase-3 (105). IL-33 activates type 2 innate lymphoid cells which induce type 2 inflammation by producing IL-5 and IL-13 (103, 106). Therefore, IL-33 is thought to be involved in the pathogenesis of AD. However, anti-IL-33 antibody LY3375880 and etokimab or anti-IL-33 receptor ST2 antibody astegolimab did not show significant clinical improvement in AD (107–109).

### 3.2 Gamma chain cytokines

Among γc cytokines, epidermal keratinocytes produce IL-7 and IL-15 (**Figure 4**, **Table 2**) (27). IL-7 is produced under the stimuli with IFN-γ (110). IL-15 expression is increased in vitiligo epidermis, and is induced by oxidative stress via NF-κB (111). IL-7 and IL-15 derived from hair follicle keratinocytes regulate skin-resident memory T cell homeostasis (112). In a mouse model of alopecia areata, blockade of IL-7 signaling with anti-mouse IL-7Rα antibody suppressed inflammatory responses and reversed alopecia areata (113). Also, in a mouse model of vitiligo, blocking IL-15 signaling with an antibody reversed the disease symptoms (114).

Epidermal keratinocytes express TSLP which is an epithelial-derived IL7-like cytokine and initiate or perpetuate the Th2-type allergic inflammation via dendritic cells or group 2 innate lymphoid cells (**Figure 4**, **Table 2**) (115, 116). TSLP mediates STAT5 phosphorylation via kinases JAK1 and JAK2 (**Table 2**) (117). The levels of TSLP is significantly increased in the lesional skin of AD, indicating that TSLP is important for initiating the systemic Th2 immunity favorable for the development of allergic inflammation (115). Against this background, a randomized phase 2a clinical trial of the anti-TSLP monoclonal antibody tezepelumab in the treatment of moderate-to-severe AD patients was conducted but did not reach the targeted level of efficacy (118).

### 3.3 IL-6 family cytokines

IL-6, the first cytokine discovered in the IL-6 family, activates the JAK-STAT pathway and induces inflammation (46, 47). Epidermal keratinocytes also produce IL-6 under the stimuli with some TLR ligands, UVB, TNF-α, IL-17, IFN-γ, and so on (**Figure 4**, **Table 2**) (119–122). Anti-IL-6 receptor antibody such as tocilizumab shows clinical efficacy in rheumatoid arthritis (RA), juvenile idiopathic arthritis (JIA), giant cell arteritis (GCA), and Castleman’s disease (CD) (123). The efficacy of tocilizumab in morphea, SSc, psoriasis, AD, vitiligo or PG has been also reported in case series, however, higher-level evidences have not been shown in these skin diseases (123).

### 3.4 IL-17 family cytokines

Epidermal keratinocytes produce IL-17C and IL-17E (IL-25) (**Figure 4**, **Table 2**). IL-17C controls the innate immune activity of epithelial cells in an autocrine manner (124). IL-17C is induced by TNF-α, IL-17A, and IFN-γ in epidermal keratinocytes (91). Anti-IL-17C antibody MOR106 showed no significant clinical improvement in AD although it was reported to be effective in mouse experiments (125, 126). IL-17E is produced by various cell types and induces Th2 responses (59). In AD, IL-17E derived from epidermal keratinocytes activates type 2 innate lymphoid cells, which drive IL-13 production (127). Therefore, IL-17E is considered to play an important role in the pathogenesis in AD. IL-17E is shown to be induced by IL-17A and IL-22 in epidermal keratinocytes (**Figure 4**, **Table 2**) (128).

### 3.5 IL-20 family cytokines

As described above, IL-19, IL-20, and IL-24 are input and output cytokines in epidermal keratinocytes (**Figures 3**, **4**, **Tables 1**, **2**). TNF-α, IL-17A, and IL-22 induces IL-19, IL-20, and IL-24 production in the cells (68, 129–131). These cytokines are considered to enhance psoriatic inflammation (130, 131).

### 3.6 type I interferons

Type I IFNs including IFN-α, IFN-β, IFN-ϵ, and IFN-κ are also output cytokines in epidermal keratinocytes (**Figure 4**, **Table 2**) (84). These cytokines are induced by TLR3 and TLR9 signaling, or Type I IFNs themselves in the cells (2, 84, 132). Since type I IFNs are considered to stimulate myeloid dendritic cells which produce IL-23 and contribute to the pathogenesis of psoriasis, randomized, double-blind, placebo-controlled, phase I study of MEDI-545 (Sifalimumab), an anti-IFN-α monoclonal antibody for plaque psoriasis was performed, however, it showed no significant clinical improvement (133). Sifalimumab was also expected to be a treatment for systemic lupus erythematosus (SLE), but the clinical trial was discontinued in favor of anifrolumab (134). Anifrolumab, a monoclonal antibody that binds to IFNAR1, therefore blocking the activity of all type I IFNs, are demonstrated to improve skin and joint disease activity in patients with SLE (134). Rontalizumab is also a monoclonal antibody, and did not show clinical efficacy including a phase 2 trial in SLE patients (134).

### 3.7 type III interferons

Among type III IFNs, IFN-λ1 is shown produced by epidermal keratinocytes stimulated with TLR3 ligand poly (I:C) or vesicular stomatitis virus (**Figure 4**, **Table 2**) (135). IFNλ and the IFNλ receptor are strongly expressed in the epidermis of cutaneous lupus erythematosus (CLE), SLE, lichen planus (LP) and dermatomyositis (135).

### 3.8 tumor necrosis factor

TNF-α is also an input and output cytokine as described above (**Figures 1**, **4**, **Tables 1**, **2**). TNF-α is induced by TNF-α itself, IL-1β, IL-17A, TLR ligands including poly (I:C), LPS, flagellin, CpG, ultraviolet light, anisomysin, palmitic acid and so on (4, 131, 136, 137).

### 3.9 Others

IL-32 is a proinflammatory cytokine which is produced by a variety of cells, including NK cells, T cells, monocytes, and epithelial cells (138, 139). IL-32 expression is increased in the epidermis of AD lesions, and the expression is induced by TNF−α and/or IFN-γ in cultured epidermal keratinocytes (139). However, IL-32 is not secreted by the cells and remains in the cells; therefore, this cytokine cannot be called an output cytokine in epidermal keratinocytes (139). This cytokine is considered to modulate keratinocyte apoptosis and contribute to the pathogenesis of AD (139).

IL-34 is an output cytokine in epidermal keratinocytes (**Figure 4**, **Table 2**). It exists as a homodimer consisting of 39 kDa monomers (140). IL-34 has no evident sequence homology with other cytokines (141). Likewise, IL-34 has only a 26% sequence homology with colony-stimulating factor 1 (CSF-1), yet they share a common receptor known as CSF-1R (**Table 2**) (141, 142). Furthermore, IL-34 has exhibits interactions with two distinct receptors: protein-tyrosine phosphatase (PTP)-ζ, and syndecan-1 (**Table 2**) (141). Through the investigation of IL-34-deficient (Il34LacZ/LacZ) reporter mice, it was found that keratinocytes and neurons were the main sources of IL-34 (143). Especially, IL-34 is highly expressed in the epidermis during murine embryogenesis (144). CSF-1R is expressed by dendritic cells (DCs) and macrophages, excluding CD11c+ precursors of DCs, whereas PTP-ζ is expressed by neural progenitors, glia, glioblastoma, B cells, and kidney tubular cells (141). Syndecan-1 is expressed by many cancers, such as myeloma, melanoma (141). IL-34 is considered to regulate major cellular functions, including cell adhesion, motility, proliferation, differentiation, survival, metabolism, and cytokine/chemokine expression (141).

IL-39 is a cytokine composed of IL-23Ap19 and Epstein–Barr virus-induced (EBI) 3 heterodimer which was firstly reported in 2015 (145). This cytokine is shown to be produced by B cell lymphocytes and activate neutrophils (146, 147). Our group researched about the expression of IL-39 in human epidermal keratinocytes, however our ELISA experiment and LC-Ms/Ms analyses did not detect the heterodimeric cytokine IL-39 in epidermal keratinocytes (148). So far, this cytokine cannot be called an output cytokine in epidermal keratinocytes **Table 3**.

**Table 3:** Disease names and the abbreviations.

Disease name	Abbreviation
Adult-onset Still disease	AOSD
Atopic dermatitis	AD
Castleman’s disease	CD
Cryopyrin-associated periodic syndrome	CAPS
Cold contact urticaria	CCU
Cutaneous lupus erythematosus	CLE
Deficiency of IL-1RA	DIRA
Familial cold autoinflammatory syndrome	FCAS
Familial Mediterranean fever	FMF
Giant cell arteritis	GCA
Generalized pustular psoriasis	GPP
Hidradenitis suppurativa	HS
Juvenile idiopathic arthritis	JIA
Lichen planus	LP
Mevalonate kinase deficiency	MKD
Muckle–Wells syndrome	MWS
Palmoplantar pustulosis	PPP
Prurigo nodularis	PN
Pyoderma Gangrenosum	PG
Rheumatoid arthritis	RA
Schnitzler’s syndrome	SS
Systemic lupus erythematosus	SLE
Systemic sclerosis	SSc
Tumor necrosis factor receptor-associated periodic syndrome	TRAPS

## 4 Conclusion

In this review, we introduced that epidermal keratinocytes recognize and produce a large number of cytokines and are deeply involved in the pathogenesis of these diseases. The number of output cytokines appears to be lower compared to that of input cytokines in the cells. This might suggest that epidermal keratinocytes are cells that are responsible for innate immunity rather than adaptive immunity, and that they are excellent at functioning as sensor cells rather than the control tower. The immunological functions of epidermal keratinocytes in innate immunity requires further investigation.

We also referred to the existence of biologics against those input and output cytokines and the target skin diseases. Current biologics have a significant impact on immune cells throughout the body, which can lead to side effects such as serious infections. If we could target only cytokines derived from epidermal keratinocytes through the development of drug delivery that specifically acts on cells, it will be possible to suppress only excessive immune reactions in the skin caused by pathological activation of epidermal keratinocytes, which should be a safer treatment.

## Author contributions

SM, YK, and KS wrote the manuscript. TM, KT, and MO contributed to writing and critically revised the manuscript. All authors contributed to the article and approved the submitted version.
